# Bleeding complications in laparoscopic cholecystectomy: Incidence, mechanisms, prevention and management

**DOI:** 10.4103/0972-9941.68579

**Published:** 2010

**Authors:** Robin Kaushik

**Affiliations:** Department of Surgery, Government Medical College and Hospital, Chandigarh, India

**Keywords:** Laparoscopic cholecystectomy, complications, bleeding, morbidity, mortality

## Abstract

**BACKGROUND:**

Laparoscopic cholecystectomy (LC) has established itself firmly as the ‘gold standard’ for the treatment of gallstone disease, but it can, at times, be associated with significant morbidity and mortality. Existing literature has focused almost exclusively on the biliary complications of this procedure, but other complications such as significant haemorrhage can also be encountered, with an immediate mortality if not recognized and treated in a timely manner.

**MATERIALS AND METHODS:**

Publications in English language literature that have reported the complication of bleeding during or after the performance of LC were identified and accessed. The results thus obtained were tabulated and analyzed to get a true picture of this complication, its mechanism and preventive measures.

**RESULTS:**

Bleeding has been reported to occur with an incidence of up to nearly 10% in various series, and can occur at any time during LC (during trocar insertion, dissection technique or slippage of clips/ ligatures) or in the postoperative period. It can range from minor haematomas to life-threatening injuries to major intra-abdominal vessels (such as aorta, vena cava and iliacs).

**CONCLUSION:**

Good surgical technique, awareness and early recognition and management of such cases are keys to success when dealing with this problem.

## INTRODUCTION

Since its introduction into the surgeon’s armamentarium, laparoscopic cholecystectomy (LC) has become the operation of choice for patients with cholelithiasis, as it is associated with lesser postoperative pain and discomfort, better cosmesis, shorter hospital stay and a chance for early return to work. However, on occasion, the procedure can be associated with serious, potentially life-threatening complications; these may arise due to injury to part of the biliary tree (biliary complications) or from procedure-related injury to other organs/ systems (non-biliary complications). Biliary complications occurring after LC have been the focus of much attention in literature, as they lead to significant morbidity (bile collections, fistulae, jaundice, cholangitis, sepsis, and other complications of bile stasis) and usually require re-operation, preferably by a specialized surgical team, to reconstruct and drain the biliary tree adequately. On the other hand, the non-biliary injuries, although reported with a variable incidence in many series and case reports, have not received their ‘due’ as complications of LC that are as dangerous and devastating as their biliary counterparts. These injuries can range from minor to major injuries of the bowel, bladder, diaphragm or intra-abdominal / biliary vasculature and have the potential to cause significant morbidity and mortality.[1]

Bleeding complications are an important subset of these ‘non-biliary’ injuries, and they can cause mortality right on the operating table if not recognized and treated in time — they are the most frequent cause of procedure-related mortality in LC (after anaesthesia-related deaths)[2,3] and yet surprisingly have not been studied comprehensively despite having been reported frequently.

## MATERIALS AND METHODS

A search was made serially through the internet (Google and PubMed) using the key words *laparoscopic cholecystectomy* AND *complications, bleeding, vascular injury, aorta, vena cava, portal vein, cystic artery and liver bed*. The results obtained were further refined to give hits only for *human* subjects, *English* language and *clinical reviews, meta-analysis and practice guidelines*. The abstracts thus obtained were scanned to ascertain their significance to the search in question, and the full texts of any such articles thus referenced were obtained. Further refinement of the results was performed by cross-referencing from the bibliography at the end of the articles that were found to be related to the subject of vascular injuries in LC.

Once this basic framework was obtained, further searches were conducted into the individual facets of such injuries, such as *laparoscopy, trocar-related complications, major-vessel injury*, and the various risk factors that have been reported in literature. The full texts of all such articles relevant to the problem being analyzed were accessed. A few texts that could not be procured were analyzed from their abstracts, and any relevant information within was included.

## RESULTS

A large volume of data exists on various aspects of LC in available English language literature (nearly 10,320 articles; Pubmed last accessed on 26^th^ April 2010), and as such, it does not seem possible that any search on any aspect of the procedure can be truly ‘complete’. However, having refined the search patterns repeatedly and having cross-referenced various studies, the results obtained do seem to be representative of the problem of bleeding encountered in relation to LC, although they should not be considered complete. The results of single-centre series of LC that have reported more than 1,000 cases in the last decade are tabulated as Table 1,[4–11] and multi-institutional studies and surveys with more than 5,000 patients analyzed are tabulated as Table 2.[12–22] Although various series have mentioned bleeding as a significant complication and a major cause of morbidity and mortality in LC, there seem to be no major reviews on this topic.

**Table 1:** Single centres (*n*> 1,000) and their reported incidence of bleeding complications during LC (last 10 years)

Author (Year)	n	Number of cases of bleeding (%)	Number of cases of major vascular injury
Khan (2010)[4]	4957	02 (0.04)	Nil
Tuveri (2007)[5]	1878	12 (0.63)	01
Marakis (2007)[8]	1225	15 (1.22)	01
Vagenas (2006)[6]	1220	19 (1.55)	Nil
Ishizaki (2006)[7]	1339	15 (1.12)	Nil
Daradkeh (2005)[9]	1208	03 (0.24)	Nil
Duca (2003)[10]	9542	224 (2.3)	01
Kaushik (2002)[11]	1233	06 (0.49)	02

**Table 2:** Multi-institutional studies and the incidence of bleeding complications during LC (*n*> 5,000)

Author (Year)	n	Number of cases of bleeding (%)
Bingener-Casey (2002)[12]	6896	44 (0.64)
Z'graggen (1998)[13]	10174	1074 (10.5)
Ihasz (1997)[14]	13833	107 (0.77)
Shea (1996)[15]	78747	163 / 15596 (1.04)
Ovaska (1996)[16]	5742	52 (0.9)
Wherry (1996)[17]	9130	149 (1.6)
Croce (1994)[18]	6865	51 (0.75)
Deziel (1993)[19]	77604	193 (0.25)
Go (1993)[20]	6076	84 (1.38)
Airan (1992)[21]	341760	17 (0.004)
Scott[22]	12397	NA

When analyzed, bleeding fell into two main groups: intra-operative and postoperative. Intra-operative bleeds occurred during the performance of the procedure, either at the time of creation of pneumoperitoneum (insertion of the Veress’ needle or first trocar) or as a consequence of improper dissection and operating technique. Essentially, intra-operative bleeding falls into one of the following four main patterns—vascular injury, slippage of clips/ligatures off the cystic artery, liver bed bleeding and miscellaneous. Although all are important, the vessel injuries are the most devastating, occurring almost exclusively during creation of pneumoperitoneum or during dissection within the Calot’s triangle. These are classified into major or minor vessel injuries.[23,24] Major vascular injury is said to occur when there is injury to the aorta, vena cava, iliac vessels, right hepatic artery or the portal vein; but when the epigastric, mesenteric and omental vessels are injured, it is considered as a minor vascular injury. As there is no definite consensus on the definition of each, a few authors have considered the mesenteric, omental, splenic, renal and even the deep epigastric vessels injuries, respectively, as major vascular injury.[25]

Postoperative bleeding is also important but poorly documented, ranging from minor haematomas to significant bleeds (missed operative injuries, slippage of clips) that require re-operation in quite a few instances. Surprisingly, very few authors have actually detailed their incidence, site and management while reviewing their data on LC. Again, it is interesting to note that there seem to be no definite criteria mentioned in literature to label a bleed as significant or major in the postoperative period.

Various factors have been implicated in the causation of vascular complications during laparoscopic surgery. These factors could be either surgeon related, patient related or related to faulty instruments and are tabulated as Table 3.[2,5,25–29]

**Table 3:** Factors implicated in the causation of bleeding complications[2,5,25–29]

Surgical Factors	Inadequate training and experience Rough technique Improper usage of instruments Inattentive handling of instruments Inadequate exposure Failure to recognize anatomical landmarks Forceful retraction
Patient-Related	Acute cholecystitis Cirrhosis Portal hypertension Coagulopathyn Adhesionss Previous surgery Anatomical abnormalities
Instrument Failures	Defective instruments

## DISCUSSION

Although LC has firmly established itself as the procedure of choice in gallbladder disease, at times, it can be associated with severe and potentially lethal complications that can be a test of patience and surgical skill even for the most experienced of surgeons. These complications can be conveniently divided into the biliary and non-biliary groups, and the majority of these are operator dependent and largely preventable. The classical injuries seen, described and taught are those of the biliary tree, and these have received much attention in literature. However, as a rule, these injuries are not immediately fatal but tend to produce ‘biliary cripples’ who suffer from all the attendant complications of cholestasis and disruption of the normal bile pathway. In contrast, the so-called ‘non-biliary’ complications, such as bleeding, bowel injury, injury to other intra-abdominal structures, or pneumothorax, have the potential for immediate fatality if not diagnosed and treated in a timely manner.

Bleeding complications account for up to one third of all major complications seen in LC,[26] and are the second most common cause of death in patients undergoing the procedure (after anaesthesia-related complications).[2,3] The reported incidence of uncontrollable bleeding in LC can be up to 2% (reported range, 0.03% to 10%),[2,13,24,25,30] but the exact figure may actually be much higher. Various factors may be responsible for this under-reporting, such as (i) lack of an exact definition of bleeding complications; (ii) many series have reported vascular injuries only but have not considered bleeding from other sites or postoperative bleeding; (iii) a publication bias; (iv) fear of litigation; and (v) absence of proper documentation at various centres. When bleeding occurs, the LC-related mortality reportedly goes up to nearly 15%, especially when the bleeding remains unrecognized.[2]

The majority of these complications are encountered in the ‘set up’ phase of the operation,[31] where the surgeon or the assistants are involved in the insertion of the trocars and the creation of pneumoperitoneum. Although the epigastric vessels are the most commonly injured site,[32,33] injuries tothe aorta and vena cava injuries are the commonest cause of mortality in such cases.[28]

## AETIOLOGY OF BLEEDING

Various factors have been implicated in literature in the causation of bleeding [Table 3].[2,5,25–29] There seems to be no single definite cause that can be established with certainty, but the surgeon-related factors are the most important, strengthening the importance of proper training and accreditation in LC. Surgeons who had operated less than 100 cases have been reported to have a higher rate of bleeding complications,[27,30] which tapered off after adequate experience had been gained; this probably represents surgeons who have had some training in their first few cases and have then started operating independently. However, this is not an absolute law, and one can face such a problem at any stage of one’s career. In fact, it has been observed that even the presence of adequate operating experience does not reduce the rate of bleeding complications, with Schafer *et al*. reporting a higher rate of complications in surgeons who had operated more than 100 cases![24] Various factors such as improper technique, inattentiveness, improper handling of instruments and inability to recognize the relevant anatomy contribute to the occurrence of bleeding complications at any level of experience. Previous abdominal surgery, anatomical aberrations, adhesions and sharp dissection may also be associated with a higher incidence of bleeding complications.

Although acute cholecystitis, cirrhosis and portal hypertension were also considered to be associated with higher complication rates and incidence of bleeding, recent reviews have suggested to the contrary; LC may actually be the procedure of choice in such patients, because of shorter operating time, lesser bleeding and lower complication rates, especially when performed by an experienced operating team.[34–37]

## PATTERNS OF BLEEDING AND MECHANISMS OF INJURY

Bleeding in LC can be encountered intra-operatively or in the postoperative period. Intra-operative bleeding usually falls into one of the following four patterns: vessel injury, slippage of clips/ ligatures of the cystic artery, liver bed bleeding and miscellaneous.

Vessel injuries are usually the most dramatic and occur either during insertion of the first trocar or during dissection/ retraction, and were rarely seen before the advent of laparoscopic surgery. The insertion of the pneumoperitoneum needle and the first trocar is considered by many to be the most dangerous step in LC, as it is essentially a ‘blind’ step of the operation. As this initial step is common to all laparoscopic operations, it has been reviewed extensively by various authors; and as mentioned earlier, the majority of bleeding complications occur in this phase of the operation.[31] Although the most commonly injured vessels are the epigastric vessels,[32,33] injury can also occur to the major intra-abdominal vessels (aorta, vena cava, iliac vessels) in 0.04% to 0.18% of patients. The distance between the abdominal wall and the great vessels can be as little as 1 to 2 cm, especially in thin individuals, contributing to the low margin of safety and the chance of aortic injury while inserting the Veress’ needle or the first trocar if due care is not taken. Aortic injury occurring at the time of surgery has also been reported while giving skin incision with the scalpel, underlining the importance of meticulous technique and care in performing each and every step of the operation. Similarly, the branching of the iliac arteries is such that the right iliac artery comes to lie just below the umbilicus, also putting it at risk of injury during forceful insertion of the trocars.[2,27,28,30,38]

Various factors have been identified in contributing to vessel injury and other trocar-related complications [Table 4],[2,26,27,29] but as always, there seems to be no substitute for adequate surgical experience. It is also important to note that no entry-technique for laparoscopy — trocar entry after creation of pneumoperitoneum, trocar entry without prior insufflation, or various modifications of the open technique of port placement — is free from complications. Although the open technique is considered by many to be safer, vascular injuries have been reported following the open technique also.[38]

**Table 4:** Risk factors for entry-related complications[2,26,27,29]

Surgical inexperience Blunt instrument Improper positioning of the patient Patient inadequately relaxed Failure to elevate the abdominal wall Incorrect direction/angle of insertion of the trocar Inadequate pneumoperitoneum Failure to rotate the trocar during insertion Forceful thrust Inability to recognize anatomical landmarks Extreme thinness Skeletal deformity Previous abdominal surgery Adhesions Multiple attempts Long trocar Small skin incisions

The risk of vascular injury is less for the secondary trocars, as they are placed under vision. However, bleeding from the abdominal wall (epigastric vessels) can be troublesome and can be avoided by transillumination of the abdominal wall and by observing penetration of the trocars through the telescope by keeping the tip of the instrument in view throughout. The inferior epigastric vessels usually lie near the lateral border of the rectus sheath, and proper placement and direction of the trocars helps in avoiding damage to them.

Dissection during LC, especially within the Calot’s triangle, can also lead to a significant bleed if the right hepatic artery or the portal vein is injured. This can happen especially when the anatomy is distorted or unrecognized, and when there is persistence in using sharp dissection in a difficult Calot’s. The right hepatic artery is more commonly injured, but the portal vein can also be injured,[39] leading to significant bleeding and the risk of biliary injury because of blind attempts to control the bleeding. Not being able to recognize the extent of injury and delaying conversion in such a situation definitely contributes to increasing the morbidity and mortality of the procedure.

Cases of bleeding because of slipped clips over the cystic artery and from the liver bed are also frequently encountered and can be troublesome enough to necessitate conversion to open procedure. Similarly, bleeding from parenchymal injuries to the intra-abdominal organs during retraction can also be the cause of much disconcert to the operating team, forcing conversion in an otherwise successful cholecystectomy.

In the postoperative period following LC, bleeding can manifest as either an internal bleeding (consequence of an intra-operatively missed vessel injury, from slipped clips/ ligatures over the cystic artery, or from the liver bed) or external bleeding (from the port sites).[24] External bleeds usually manifest after surgery, with soakage of dressings or visible bleeding from port sites, and may require reoperation to salvage patients who do not respond to conservative measures. For patients having internal bleeding, it is difficult to establish the site of bleeding, and such patients may need re-exploration for its control. Surprisingly, despite there being a large volume of data on LC in literature, not much is available on the incidence of postoperative bleeding after LC, the indications for operating such patients, and the operative findings. Persisting pain, tachycardia, fall in haemoglobin and blood pressure and obtundation should alert one to the possibility of bleeding even in the absence of external bleeding, and these patients need to be evaluated carefully for their response to conservative means and the need for surgery.

## CLASSIFICATION OF BLEEDING COMPLICATIONS

There is a lack of uniformity in the classification of bleeding complications of LC, with various authors arbitrarily defining major and minor vascular injuries. Many have not even considered the other sites of bleeding that have been reported in literature. A working classification as proposed by Schafer *et al*.[24] divided bleeding complications during laparoscopy into *intra-operative* (local haemorrhage into the peritoneal cavity, retroperitoneum or abdominal wall) and *postoperative* (bleeding within 24 hours of surgery) bleeding complications. Each was further divided into external bleeding (abdominal wall) and internal bleeding (peritoneal cavity, retroperitoneum). Major vascular injury was defined as injury to any of the following vessels: aorta, vena cava, iliac vessels, mesenteric vessels, portal vein, and splenic, omental and renal vessels. However, they did not define what constituted a minor vascular injury, and their definition differed from that adopted by Shamiyeh (epigastric, mesenteric and omental vessels considered to be minor vascular injuries).[23] Nordestgaard, however, felt that injury to the deep epigastric vessels should be considered a major-vessel injury, as it had the potential to cause significant morbidity and mortality.[25]

Standardization of the definitions of bleeding in relation to LC may help in defining the exact incidence of such complications in a better way, and a suggested simple classification system is shown in Table 5, wherein the bleeding complications are divided into major and minor depending on the need for conversion, additional surgical procedures, or blood transfusions. Thus, any bleeding that requires a laparotomy is major, irrespective of the vessel injured or the timing (intra-operative or postoperative). Similarly, any bleeding that needs additional surgical procedure (wound exploration and ligation of bleeder) or blood transfusion is also taken to be major, whereas bleeds controllable by pressure, packing; or abdominal wall haematomas that do not require any additional manoeuvres can be classified as minor bleeds.

**Table 5:** Suggested classification of the patterns of bleeding complications in laparoscopic cholecystectomy

	Major	Minor
Intra-Operative Bleeding	Any bleeding that requires conversion for control/repair. This could be — Bleeding from intra-abdominal vessels: Aorta Vena cava Superior mesenteric vein Portal vein Right hepatic artery Cystic artery Mesenteric vessels Omental vessels	Bleeding from the vessels of the abdominal wall: Epigastric vessels — have the potential to cause significant haemorrhage but are by and large controllable by pressure, packing or suturing
	Bleeding from any other site such as	
	The liver bed	
Postoperative Bleeding	Any bleeding, external or internal, that requires Re-exploration Additional surgical procedure such as wound exploration Blood transfusion	Abdominal wall haematomas Port site bleeding that can be controlled without additional surgical means beyond pressure, packing or suturing

## DIAGNOSIS AND MANAGEMENT

The diagnosis of vascular injury may be straightforward and obvious, but it may be missed if not suspected and carefully looked for. Blood coming out through the Veress’ needle is a sure indication of puncture of a major vessel, and time must not be lost in converting to a formal laparotomy to assess the nature of injury and salvage the patient. In such a situation, it is recommended not to withdraw the needle but to let it remain in place while performing the laparotomy — not only does the needle tamponade the bleeding, it also serves as a guide to the exact site of injury.[40]

However, at times, there may be no obvious bleeding even when major vessels are involved, since the retroperitoneal vessels do not tend to produce free intraperitoneal bleeding. In addition, the high intra-abdominal pressures due to pneumoperitoneum may reduce the arterial bleeding, leading to these injuries being missed initially.[1] As a rule, a generalized laparoscopy must be performed after insertion of the first trocar, and the presence of a retroperitoneal or expanding haematoma near the site of insertion should alert one to the possibility of vessel injury. The onset of unexplained haemodynamic instability and fall in end tidal carbon dioxide after insertion of the Veress’ needle or first trocar should also make one think of this possibility even if there is no obvious bleeding.[1,2,26,29]

Where faced with the possibility of injury to the aorta, vena cava or the iliac vessels, conversion to open surgery must be quick. It is in the interests of the patient if another consultant or colleague can be asked to join the surgery as soon as possible. The basic principles underlying the management of major-vessel injuries are, prompt control (packing, digital pressure, vascular clamps), isolation, dissection and repair.[25,40,41] Once the vessel has been identified and dissected, care must be taken to look at the posterior wall also to rule out a ‘through and through’ penetration injury.[40] One must also always look for other associated injuries, injury to other vessels and injury at multiple sites on the same vessel.[40,41] Repair is usually straightforward, with primary repair being considered ideal, although ligation of the iliac veins has also been performed safely; however, if the damage is extensive, some form of grafting may be required.[41] If the patient remains stable, one can then proceed with cholecystectomy; but in a patient who has bled extensively and is unstable, the procedure may have to be postponed.
